# An Age-Related Hearing Protection Locus on Chromosome 16 of BXD Strain Mice

**DOI:** 10.1155/2020/8889264

**Published:** 2020-06-08

**Authors:** Qing Yin Zheng, Lihong Kui, Fuyi Xu, Tihua Zheng, Bo Li, Melinda McCarty, Zehua Sun, Aizheng Zhang, Luying Liu, Athena Starlard-Davenport, Ruben Stepanyan, Bo Hua Hu, Lu Lu

**Affiliations:** ^1^Department of Otolaryngology-Head and Neck Surgery, Case Western Reserve University, Cleveland, Ohio, USA; ^2^Hearing and Speech Rehabilitation Institute, College of Special Education, Binzhou Medical University, Yantai, China; ^3^Department of Genetics, Genomics and Informatics, University of Tennessee Health Science Center, Memphis, TN 38106, USA; ^4^Department of Neurosciences, Case Western Reserve University, Cleveland, Ohio, USA; ^5^Center for Hearing and Deafness, University at Buffalo, NY 14214, USA

## Abstract

Inbred mouse models are widely used to study age-related hearing loss (AHL). Many genes associated with AHL have been mapped in a variety of strains. However, little is known about gene variants that have the converse function—protective genes that confer strong resistance to hearing loss. Previously, we reported that C57BL/6J (B6) and DBA/2J (D2) strains share a common hearing loss allele in *Cdh23*. The cadherin 23 (*Cdh23*) gene is a key contributor to early-onset hearing loss in humans. In this study, we tested hearing across a large family of 54 BXD strains generated from B6 to D2 crosses. Five of 54 strains maintain the normal threshold (20 dB SPL) even at 2 years old—an age at which both parental strains are essentially deaf. Further analyses revealed an age-related hearing protection (*ahp*) locus on chromosome 16 (Chr 16) at 57~76 Mb with a maximum LOD of 5.7. A small number of BXD strains at 2 years with good hearing correspond roughly to the percentage of humans who have good hearing at 90 years old. Further studies to define candidate genes in the *ahp* locus and related molecular mechanisms involved in age-related resilience or resistance to AHL are warranted.

## 1. Introduction

Age-related hearing loss (AHL), or presbycusis, is a major sensory impairment [1] generally caused by the degeneration of hair cells within the organ of Corti [2]. AHL is characterized by a slow progressive decline in hearing sensitivity and balance [3]. AHL can contribute to social isolation, depression, and even cognitive decline [4, 5]. Approximately 35% of adults between 65 and 75 years old have some degree of hearing loss; and by 75 years, 40–50% have AHL. Like most age-related neurodegenerative diseases, AHL is genetically complex due to interaction with many environmental risk factors (e.g., noise, smoking, ototoxic drugs, and disease). Due to its late onset, genetic analysis is difficult [6, 7]. The use of inbred mouse models can provide an ideal translational bridge to study AHL and to enable mechanistic and preclinical therapeutic studies aimed at devising new treatments.

Inbred strains of mice have been effectively used to investigate AHL [8, 9]. We and others have mapped several quantitative trait loci (QTLs) in a variety of inbred mice, including *ahl* [10, 11], *ahl2* [12], *ahl3* [13, 14], *ahl4* [15], *ahl5*, and *ahl6* [16], that greatly increase the risk of AHL. The *ahl* locus is now known to be a mutation in *Cdh23* [17, 18]. Both C57BL/6J (B6) and DBA/2J (D2) strains of mice are homozygous for the *Cdh23^c.753A^* allele and have progressive hearing loss [10]. However, the D2 strain exhibits a much early-onset of hearing loss, starting from 3 weeks old, and most animals are deaf by 3 months [8, 19, 20]. In contrast, B6 mice only develop high-frequency hearing loss starting at 3 months old, and the loss progresses to low-frequencies and worsen to a profound level only by 12 months [8, 10, 11, 21].

Recombinant inbred (RI) strains have been widely used in genetic mapping and studies of gene-gene, gene-environment, or gene-drug interactions, as well as gene expression-molecular pathways for Mendelian and quantitative traits [22, 23]. Conventional mouse RI strains are developed by crossing two inbred parental strains and repeatedly mating the resulting siblings for 20 generations or more to ensure that they are at least 99% inbred [24].

We conducted this study using BXD RI strains derived from crosses between B6 and D2 parents that are commonly used as models of AHL. BXDs are a very large mouse family (*N* = 152), which is optimal to replicate experiments across different laboratories or at different years as long as they have the same substrain name. At the same time, the diversity among the 152 strains offers a very powerful tool for mapping and analyzing the genetic origin of complex traits, such as hearing loss [25, 26]. The BXDs are an unrivaled resource for auditory system genetics because the parental strains are suitable hearing loss models with a significant difference in age-related progressions. Additionally, both parental strains and all of their highly diverse BXD RI progeny have been well-sequenced, and more than 6 million sequence variants have been identified and segregated among the BXD strains [27]. Data for investigating the BXD family is available on our open-source database (http://www.genenetwork.org), which is now widely used as an experimental platform for personalized and probabilistic medicine.

## 2. Materials and Methods

### 2.1. Mice

We completed a hearing screen of 2–5 cases for each of 54 BXD strains plus B6 and D2 parental strains in total 170 mice. All were between 12 and 32 months old when tested. Animals were housed and maintained on a 12 : 12 light/dark cycle, with *ad libitum* access to food and water. All experimental procedures were in accordance with the *Guidelines for the Care and Use of Laboratory Animals* published by the National Institutes of Health and were approved by the Animal Care and Use Committee at the University of Tennessee Health Science Center (UTHSC; Memphis, TN, USA).

### 2.2. Hearing Screening

Hearing acuity was assessed using an auditory-evoked brainstem response (ABR) test [8]. All the hearing evaluation was performed at UTHSC (University of Tennessee Health Science Center) by Dr. Zheng, who has experience in testing the ABR in over ten thousand mice. In brief, the mice were anesthetized with an intraperitoneal injection (IP) of ketamine, xylazine, and acepromazine at doses of 40, 5, and 1 mg/kg, respectively. The body temperature was maintained at 37–38°C. ABR testing was carried out using a SmartEP system from Intelligent Hearing Systems (Miami, FL). The ABRs were recorded using platinum subdermal needle electrodes inserted at the vertex (active electrode), ventrolateral to the right (reference electrode) and left (ground electrode) ears. The acoustic stimuli were tone-bursts (3 ms duration with a 1.5 ms cosine–gated rise/fall time) that were delivered through a high-frequency transducer (closed system). The tone-bursts were delivered to both ears simultaneously, and the recorded responses represented the threshold of the better hearing ear. The stimuli were presented in a 5 or 10 dB step decrement from 70 dB SPL until the lowest intensity that could still evoked a reproducible ABR pattern was detected. Average ABR thresholds for mice with normal hearing were about 30, 20, and 45 dB SPL for 8, 16, and 32 kHz tone bursts, respectively. In the current study, we defined the threshold shift of 20–40 dB SPL as mild impairment, 41–60 dB as intermediate impairment, and greater than 60 dB as profound impairment [12].

### 2.3. Examination of Morphological Phenotype

Cross-sections for hematoxylin-eosin (H&E) staining and whole-mount basilar membrane surface preparations were performed to define the site and the level of cell damage. The inner ears from mice were collected, perfused with Bouin's fixative, then left immersed in fixative for 48 h, decalcified with Cal-EX solution for 6 h, and embedded in paraffin. Tissue sections of 5 *μ*m were cut, mounted on glass slides, and stained in H&E. The stained tissues were observed under a light microscope. We performed morphological analyses of six BXD strains with either extremely good or poor hearing.

### 2.4. Heritability Estimation of Hearing Phenotypes

We estimated the narrow heritability of three hearing phenotypes (8, 16, and 32 kHz stimuli) with the following equation [28], in which variances among strain means were compared to the total variance.

(1) $$\begin{array}{c} h^{2}=\frac{0.5VA}{0.5VA+VE} \end{array}$$

VA is the variance among strain means, and VE is the variance within strains.

### 2.5. Mapping of Auditory Acuity Loci

One of the primary uses of the BXD family is to map QTLs that modulate hearing phenotypes of the auditory system [29]. All ~7200 BXD informative genetic markers (http://www.genenetwork.org/webqtl/main.py?FormID=sharinginfo&GN_AccessionId=600) were checked for association with each hearing phenotype at 8, 16, and 32 kHz. This analysis was done using the WebQTL tool on our GeneNetwork website (http://www.genenetwork.org) [30, 31]. The likelihood ratio statistics (LRS) score computed with the Haley-Knott equations [32] was used to evaluate linkages between differences in traits and differences in particular genotype markers. Genome-wide significance (*p* value < 0.05) was calculated based on 1000 permutation tests. The phenotype-associated QTLs using the Haley and Knott method were further confirmed with GEMMA, a linear mixed model mapping algorithm that accounts for kinship among the BXD strains. For GEMMA mapping results, 4 LOD score (equal to –log(*p*) of 4) was set to the genome-wide significant threshold. The confidence interval was estimated by a 2 LOD drop-off method [33].

### 2.6. Variant Identification

Sequence differences segregating the BXDs have been described in a previous work [34]. In this study, we have focused on variants that change protein sequence, such as nonsense, missense, and frameshift mutations.

### 2.7. Gene Expression Resource

Gene expression levels of the inner ear for the genes within the QTL interval were explored at the gEAR portal online resource (https://umgear.org/). The gEAR portal is a website for visualization and analysis of multiomic data both in public and private domains. In addition, the gEAR portal enables upload, visualization, and analysis of single-cell RNA sequencing data (scRNA-seq data).

### 2.8. Data Analysis

Data Desk 8.1 software was used to calculate means, SD, and variance. Differences between two groups (such as two groups of strains with extremely good and poor hearing) were analyzed using a two-tailed Student's *t*-test.

## 3. Result

### 3.1. D2 Mice Exhibit an Early-Onset Hearing Deficit and Associated Loss of Spiral Ganglion Neurons and Hair Cells

**Figure 1:**
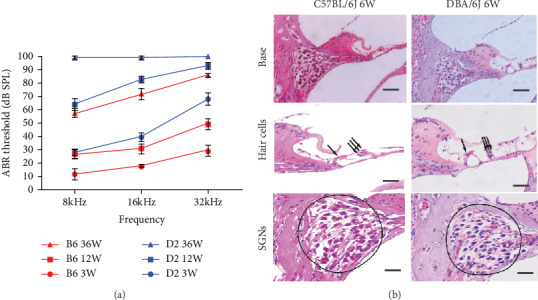
(a) ABR thresholds of three age groups in parental D2 and B6 strains (*n* = 5 for each group). ABR thresholds of D2 and B6 mice were determined at three frequencies shown on the *x*-axis. Error bars indicate the standard error of the mean (SEM). (b) Cross-sections (5 *μ*m) through the modiolus of the cochleae from a D2 and a B6 mouse at the age of 6 weeks. Overall morphological characteristics of the basal turn (scale bars = 200 *μ*m) and degeneration of spiral ganglion cells and hair cells (scale bars = 50 *μ*m) were observed.

We assessed the hearing sensitivity of B6 and D2 parental strains by ABR threshold measurements. Both B6 and D2 mice displayed progressive hearing loss. Loss of hearing in D2 mice occurred much earlier and was more profound than that in B6 mice. The D2 strain began to exhibit hearing loss as early as 3 weeks old, and the loss progressed to a severe level within 2–3 months. The B6 mouse strain showed hearing loss starting at 3 months old, and the loss progressed to a wider frequency range and a profound level after 9 months (Figure 1(a)). Sections of the cochleae from B6 and D2 mice were examined microscopically for an initial gross assessment of cochlear pathology. Hearing loss in both B6 and D2 mice was accompanied by progressive degeneration of the organ of Corti and spiral ganglia. The SGNs began to lose at the age of 6 weeks in D2 when hearing loss developed (Figure 1(b)).

### 3.2. The Hearing Threshold Is a Gradient Distribution in BXD Strains

**Figure 2:**
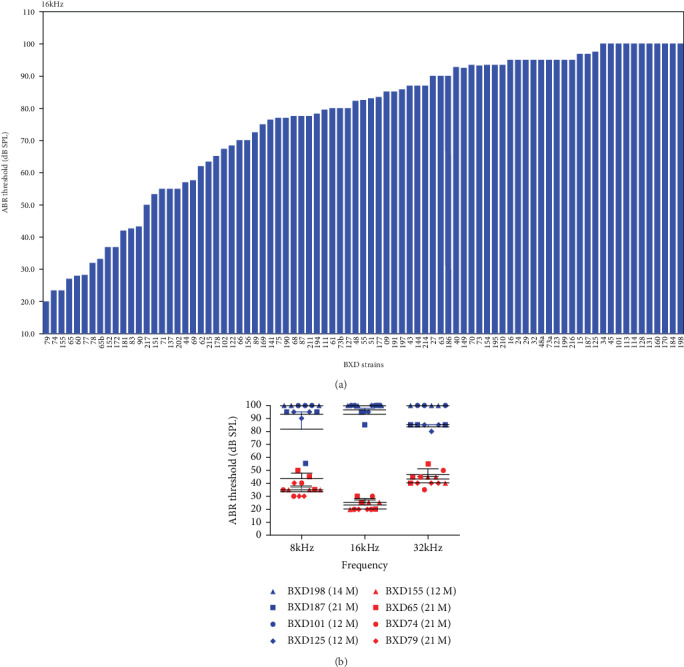
(a) The ABR thresholds of 54 BXD mouse strains at 2 years old display a gradient distribution at 16 kHz. Other frequencies have also been tested (data not shown). (b) ABR thresholds of good-hearing (BXD79, BXD74, BXD65, and BXD155) and poor-hearing (BXD198, BXD187, BXD101, and BXD125) mice are exemplified, respectively. Each data point represents an average threshold value (calculated as the arithmetic mean) for each age group (*n* = 3) described in the adjacent data point legend. Error bars indicate the standard deviation from the SEM. The ABR thresholds were also statistically analyzed, showing significant differences between the four good-hearing and four poor-hearing strains (*p* < 0.05 by ANOVA test).

We screened 54 BXD strains (aged 12–32 months) for hearing loss at 8, 16, and 32 kHz. The heritability was around 50–70% for the 3 frequencies, suggesting that genetic factors significantly affected hearing loss with aging. The threshold level was found to be a gradient distribution in BXD strains (Figure 2(a)). The results showed that the thresholds of the three tested frequencies varied significantly among BXD strains. The 16 kHz measurement showed that some BXD strains (including BXD79, 155, and 74) had a favorable hearing threshold measured at 21 months, while others (including BXD198, 101, and 45) had severe hearing loss (100 dB SPL) at 1 year old (Figure 2(b)). We hypothesize that this gradient distribution of hearing in BXD strains is due to the segregation of genes carried by D2 and B6 inbred strains.

### 3.3. Several BXD Strains Retain Excellent Hearing at the Age of Two Years

**Figure 3:**
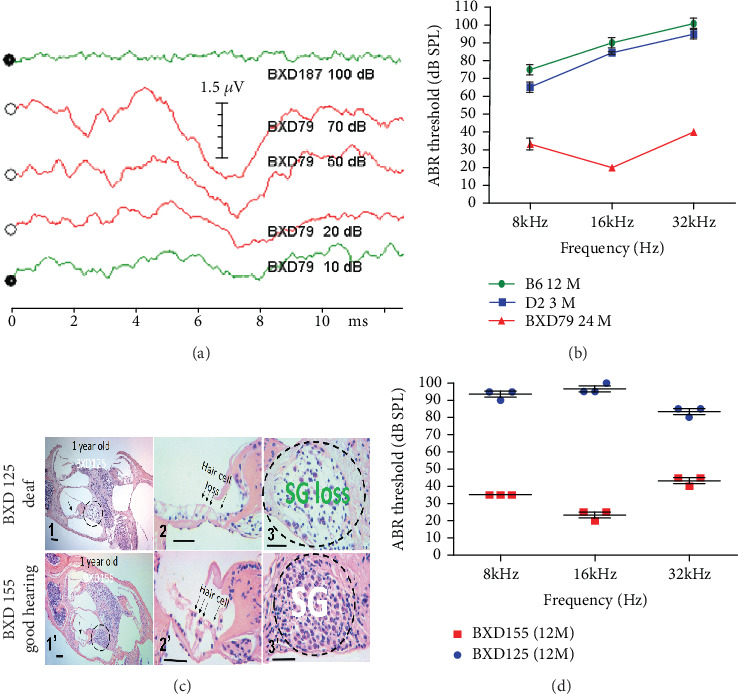
(a) 16 kHz ABR testing of a good-hearing BXD79 mouse revealed measurable responses to sound as low as 10 dB (bottomed greenline) and a deaf BXD187 mouse had no response to 100 dB (the top green line); both mice are at the age of 21 months. (b) ABR thresholds of parental 3-month-old D2 mice, 12-month-old B6 mice, and 24-month-old BXD79 mice that have the best hearing among the 54 examined BXD strains. (c) H&E stained cross-sections of the inner ears from a deaf mouse (BXD125) (C1-3) and a good hearing mouse (BXD155) (C1′-3′). Morphological contrast of the whole cochleae from the deaf BXD125 (C1) and the good hearing BXD155 (C1′) strain. OHCs and IHCs are lost in the deaf ear (C2) but are present in the good ear (C2′). Spiral ganglion (SG) cells are lost in the deaf ear (C3) but are present in the good ear (C3′). Scale bars = 50 *μ*m. The corrected densities of the SGNs (420 ± 28) in the basal cochlear turn in deaf BXD mice (*n* = 3) are significantly less than those of good hearing BXD mice (1411 ± 39) (*n* = 3; *p* = 0:0001 by *t*-test). No significant differences in the mean density of the SGNs in the apical cochlear turns were observed (data not shown). (d) ABR thresholds of deaf BXD125 and good-hearing BXD155 mice are exemplified at 1 year old (*n* = 3). Error bars indicate the standard deviation from the SEM. The ABR thresholds are significantly different between the two strains (*p* < 0:05 by ANOVA test).

Our ABR assessments revealed that several BXD strains retained excellent hearing at the age of two years (Figure 2(a)). For example, the 16 kHz threshold in BXD79 remained at the level of 20 dB SPL at the age of 21 months (Figure 3(a)). We observed that the D2 strain began to exhibit hearing loss at 3 weeks old which progressed to severe hearing loss within 2–3 months. B6 mice had a normal hearing before 3 months old, and then their hearing began to decline starting from high frequencies (32 kHz) as we measured. The level of hearing loss among three-month-old D2 and 12-month-old B6 mice was more severe compared to the 2-year-old BXD79 strain (Figure 3(b)). We performed H&E staining of cross-sections of the inner ear for 3 strains with partial hearing loss and 3 strains with deafness at 1 year old. We observed a loss of the inner hair cells (IHCs) and outer hair cells (OHCs) and a decrease in the density of SGNs in the strains with deafness (Figures 3(c) and 3(d)).

### 3.4. A Novel Age-Related Hearing Protection (*ahp*) Locus Maps to Chr 16

**Figure 4:**
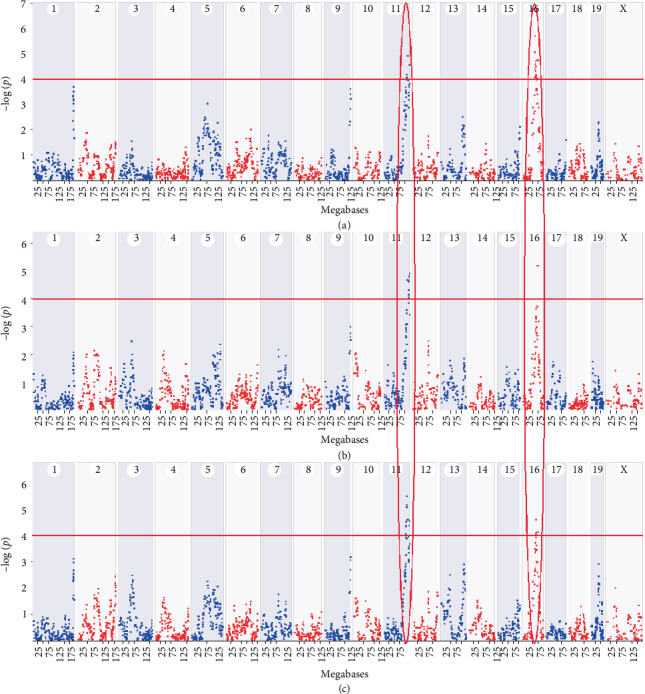
Manhattan plot of linkage to hearing phenotypes. QTL mapping identified one novel QTL on Chr 16 and one known QTL on Chr 11 for all three hearing phenotypes at 8, 16, and 32 kHz (a–c). The *x*-axis denotes a position on the mouse genome, in megabases (Mb), while the *y*-axis gives the –log(*p*) of linkage. The red line indicates a significant threshold for a genome-wide scan at a LOD score of 4 (equal to –log(*p*) of 4). Maps were computed with GEMMA using LOCO option. All analyses were performed on GeneNetwork.

One novel QTL for all three frequencies was identified on Chr 16 at 69.6 Mb, with a peak LOD score of 5.7, 5.2, and 4.6 for 8, 16, and 32 kHz, respectively (Figure 4). This QTL encompasses 19 Mb from 57 to 76 Mb. However, this novel QTL was unable to be detected when we performed QTL mapping by excluding several strains with relatively good hearing (ABR < 35 dB SPL), which suggests that including mice with a good hearing in the study is obligatory in discovering novel QTL associated with hearing protection. In addition, we identified a QTL on Chr 11 significantly associated with all three frequencies, with peak LOD score of 4.9, 4.9, and 5.5 for 8, 16, and 32 kHz, respectively (Figure 4) This QTL overlapped with the previously identified AHL locus *ahl8* [20]. The *ahl8* was proven to be a nonsynonymous variant (rs26996001) of *Fscn2* gene in D2 mice, which causes an amino acid change from arginine to histidine at position 109 (R109H) [35]. The wild-type allele of this mutation prevents hearing loss. Loss of function of this gene has been shown to cause a variety of morphological and functional changes, including malformation of stereociliary bundles of cochlear hair cells, abnormal outer hair cell physiology, abnormal ABR, abnormal distortion product otoacoustic emission, a decrease in the numbers of stereocilia in both inner and outer hair cells, an increase in the susceptibility to AHL, short cochlear hair cell stereocilia, and outer hair cell degeneration [35, 36].

### 3.5. Exploration of Candidate Genes in QTL Region of Chr 16

The QTL region at Chr 16 harbors 145 genes, including 67 protein-coding genes, among which 25 are olfactory receptor genes (Table 1). The remaining genes are predicted genes or pseudogenes.

We explored whether genes in the QTL region harbored protein-altering variants, which could be responsible for the generation of observed hearing phenotype. With our previously sequenced whole genome sequences of D2 and B6, we identified 10 genes that harbor nonsynonymous variants, including *Arl6*, *Crybg3*, *Epha3*, *Epha6*, *Filip1l*, *Gabrr3*, *Gbe1*, *Gpr15*, *Hspa13*, and *Samsn1* (Table 1). In addition, *Htr1f* contains a frameshift variant. No protein-altering variants were found within the other genes in the QTL interval.

Next, we explored whether the genes in the QTL region, especially for those protein-coding genes, were expressed in the hearing relevant tissue or cell types. By searching the gEAR portal (https://umgear.org/), a database for gene expression analysis resource, we found 19 genes that are highly expressed in hair cells, epithelial nonhair cells, or the cochlear duct, including *Tomm70a*, *Nit2*, *Tbc1d23*, *Tmem30c*, *Col8a1*, *Dcbld2*, *Cpox*, *Crybg3*, *Arl6*, *Arl13b*, *Pros1*, *Epha3*, *Zfp654*, *Cggbp1*, *Chmp2b*, *Vgll3*, *Robo1*, *Robo2*, and *Hspa13* (Table 1). In addition, 11 genes have a relatively low level of expression, including *Filip1l*, *St3gal6*, *Gpr15*, *Gabrr3*, *Epha6*, *Nsun3*, *Stx19*, *Htr1f*, *Cadm2*, *Gbe1*, and *Rbm11*.

Based upon information, including gene mutation and expression, we categorized QTL candidates into three layers (Table 1): top priority, median priority, and low priority. Top priority candidates included genes with functional mutation and highly expressed in cochlear hair cells, epithelial nonhair cells, or the cochlear duct. Median priority candidates included the followings: (1) genes with functional mutation and expressed in the hearing relevant tissue; and (2) genes with high expression in the hearing relevant tissue, but no functional variants. The rest of the candidates were defined as Low priority candidates.

It is worth noting that two genes within this locus have been implicated in the hearing function: *Arl6* (ADP-ribosylation factor-like 6) and *Pou1f1* (POU domain, class 1, transcription factor). *Arl6* has been linked to both sensorineural and conductive hearing impairment, while *Pou1f1* is associated with the abnormal orientation of outer hair cell stereociliary bundles, and abnormal morphology in outer hair cells, stria vascularis, and the tectorial membrane in the cochlea. *Pou1f1* is functionally associated with a decreased endocochlear potential, the absence of cochlear microphonics and distortion product otoacoustic emissions, and deafness [37].

## 4. Discussion

We have previously mapped several hearing loss QTLs, such as *ahl* [10], *ahl2* [12], and *ahl4* [15]. We also located *ahl8* [20] within 32 BXD strains from the Jackson Laboratory. A QTL locus underlying the early-onset, low-frequency hearing loss in BXD strains has been mapped at chromosome 18, *ahl9* [38]. All of these previous data were collected from mice younger than one-year-old. The primary objective of previous studies was to identify variants that increase the risk of hearing loss. At present, only a limited number of deafness-resistant QTLs have been mapped. Thus, studies designed to identify genetic variants that protect from hearing loss are critical.

The BXD strains are currently the largest and best phenotyped genetic reference population. The genomes of both parental strains have been extraordinarily well-sequenced, giving us the information on essentially all sequence variants that segregate [26] among BXD strains. Most importantly, D2 and B6 are commonly used mouse models of AHL loss. We observed that hearing loss in D2 mice occurs much earlier than does the B6 as illustrated by our functional analysis with ABR. By 12 months, both B6 and D2 mice displayed massive hearing loss with D2 mice having even greater pathogenesis (see Figure 1 and our previous reports [8, 39, 40, 41, 42]). These features are quite similar to those of human presbycusis in which hearing loss starts from higher frequencies, followed by middle- and lower frequencies. The functional loss is associated with degenerative changes in highly energetic cells of the *stria vascularis*, spiral ganglion neurons, and cochlear hair cells, especially outer hair cells. Cells of the *stria vascularis* generate the endocochlear potential (EP; also called endolymphatic potential), a positive voltage of 80-100 mV in the cochlear endolymphatic space [43, 44]. Notably, although D2 mice functionally have much early and severe hearing loss, histologically, the loss of spiral ganglion cells and hair cells at 6 weeks old did not differ much from B6 mice, suggesting that the unique genetic background in the D2 strain offers protection of spiral ganglion and hair cell bodies but not stereocilium tips where mutant fascin-2 disturbs crosslink function and slows actin depolymerization at stereocilium tips that are important for maintaining the stereocilium length as previously proposed [36].

The BXDs are a logical and powerful first resource for the systems genetics analysis of hearing loss. We have screened 2-5 mice per strain across 54 BXD strains and confirmed QTL on Chr 11 where *Fscn2* is identified as a causal gene of hearing loss [35] and found 1 novel QTL on Chr 16 in aged BXD mice that is most likely an *ahp* locus in BXD strains.

The hearing threshold has a gradient distribution in BXD strains (see Figure 2). Several BXD strains have remarkably intact hearing even at 2 years old—an age at which both parental strains are essentially deaf (Figure 3(b)). ABR thresholds of good-hearing BXD79 mice at 21 months old are much better than those of BXD187 mice at 12 months old (*p* < 0.05, see Figure 2(b)). This is mainly because the traits and genes of the parental strains begin to segregate. In previous studies, D2 mice have the early onset of progressive hearing loss with mutations in several genes known to cause hearing loss, including *Cdh23^c.753A^* and *Fscn2^R109H^* [10, 35]. The features of deafness in D2 mice segregate among BXD family members. The B6 mice share the *Cdh23^c.753A^* mutation with D2 [11, 19], having mid-aged hearing loss. The features of hearing protection also segregate among BXD family members. Both D2 and B6 mice carry *Cdh23^c.753A^*, and both are nearly deaf over a year old. All BXD strains should carry the *Cdh23^c.753A^*. Why do a few BXD strains maintain good hearing even around 1-2 years old? We hypothesize that BXD strains harboring the protective locus preserve better hearing at older ages.

As demonstrated in previous studies and our current investigation, hearing loss in B6 and D2 mice are accompanied by degeneration of the organ of Corti and spiral ganglia (see Figure 1(b)) [19, 45]. This degeneration is caused mainly by *Cdh23* [17, 18, 46]. Our histological examination of the one-year-old BXD strains revealed a highly correlated change in hearing dysfunction and cochlear pathogenesis. Hair cell loss and spiral ganglia loss happened earlier and more severely in the basal turn of the cochlea, which corresponds to high-frequency hearing loss exhibited in both parental strains and the vast majority of BXD strains. Taken an example from the 5 best hearing BXD strains out of the 54 total strain so far we have tested, the good hearing strain (responded well to as low as 10-dB tone bursts, see the bottom green sweep in Figure 3(a)) corresponded to the nearly intact histological structures (1′, 2′, and 3′ in Figure 3(c)). In contrast, the deaf strain (the top sweeps in Figure 3(a)) corresponded with the severe loss in hair cells and spiral ganglion cells (1, 2, and 3 in Figure 3(c)). The hearing threshold between the two strains showed a statistically significant difference (Figure 3(d)). These dramatic contrasts, both in hearing function and pathology, have not been reported in any BXD strains at such old age. This suggests that the protective loci in BXD strains interact with *Cdh23* to reduce the damage to the auditory system.

QTL analysis was used in our current research. We screened 54 aged BXD strains and performed a QTL analysis for hearing phenotypes at 8, 16, and 32 kHz. This analysis unveiled one novel QTL on chromosome 16 (Chr 16) and one known QTL on chromosome 11 (Chr 11) for all 3 measurements (Figure 4). Nevertheless, the novel QTL on Chr16 is completely undetectable when we remove the mice with the ABR threshold <35 db SPL (6 strains with the best hearing, 11%), while Chr 11 QTL (*alh8*) shows enhanced signals at all 8, 16, and 32 kHz. We and others have reported that the locus on Chr11 (*ahl8*) contributing to progressive hearing loss in D2 mice is a missense variant of the *Fscn2* gene [20, 35]. The *Cdh23^c.753A^* mutation is shared by both D2 and B6 [10], and it is consistent with our QTL analysis of 3 measurements. A total of 155 genes are mapped to the 73.6 Mb interval of Chr 16; however, only 67 are protein-coding genes that include 25 olfactory receptor genes (Table 1). The rest of them are either predicted genes or pseudogenes. After filtering based on the protein-altering variants, the best candidate genes for Chr 16 are *Arl6*, *Crybg3*, *Epha3*, *Epha6*, *Filip1l*, *Gabrr3*, *Gbe1*, *Gpr15*, *Hspa13*, and *Samsn1*. *Htr1f* is identified as a frameshift variant.

MSM derived Ahl3 in B6-Chr17 (MSM) consomic mice showed a prominent hearing loss resistance and was mapped on chromosome 17 in 2004 [13]. Apparently, identifying candidate genes in this locus will significantly help us understand age-related hearing protection at the molecular level and potentially help to define therapeutic drug target for preventing human presbycusis. But so far, the Ahl3 has not been defined at the gene level because the disadvantage of the whole chromosome 17 substitution makes the genetic mapping impossible for narrowing down. Thus, we should take the advantages of many strains of BXD with each small segment of every chromosome that has reshuffled for over 20 generations. Nevertheless, RNA-seq data from the inner ear of BXD strains are needed for further investigation of the *ahp* at the gene and molecular levels.

Many of our commonly used hearing loss inbred mouse models carry the *Cdh23^c.753A^* allele [10, 47], which are relatively numerous in our studies in mouse models. Mutations of the *Cdh23* gene are involved in a spectrum of hearing impairments, including hearing loss with vision loss, Usher syndrome 1D, early-onset progressive hearing loss, and AHL in humans or mouse models. Two recent studies have shown that *Cdh23* mutations significantly contribute to AHL in humans [47, 48]. As a result, *Cdh23* is an important gene linked to hearing loss. Thus, the protective locus may prevent hearing loss in humans.

In summary, using QTL mapping, we have identified a novel locus on Chr 16 that is a significant contributor to the protection of hearing. It lessens the decline of the auditory function and pathology related to AHL, a disease affects the quality of lives in more half of the elderly over 75 years old. The discovery of this locus will help to better understand the molecular mechanism of AHL and provide clues for identifying new candidate genes responsible for human senile deafness and other hearing impairment.

## Figures and Tables

**Table 1:** List of the protein-coding genes within the QTL region of Chr 16.

Entrez ID	Symbol	Location (Chr and Mb)	Distance to QTL peak (Mb)	Variant	Expression^∗^	Candidates rank
224273	Crybg3	16 : 59.490775	-10.11	Nonsynonymous SNP	High	Top
56297	Arl6	16 : 59.613321	-9.99	Nonsynonymous SNP	High	Top
13837	Epha3	16 : 63.545218	-6.05	Nonsynonymous SNP	High	Top
110920	Hspa13	16 : 75.75519	6.16	Nonsynonymous SNP	High	Top
78749	Filip1l	16 : 57.353277	-12.25	Nonsynonymous SNP	Low	Median
71223	Gpr15	16 : 58.717435	-10.88	Nonsynonymous SNP	Low	Median
328699	Gabrr3	16 : 59.407382	-10.19	Nonsynonymous SNP	Low	Median
13840	Epha6	16 : 59.641433	-9.96	Nonsynonymous SNP	Low	Median
15557	Htr1f	16 : 64.924729	-4.68	Frameshift	Low	Median
74185	Gbe1	16 : 70.313949	0.71	Nonsynonymous SNP	Low	Median
67742	Samsn1	16 : 75.858794	6.26	Nonsynonymous SNP	Low	Median
28185	Tomm70a	16 : 57.121714	-12.48	NA	High	Median
52633	Nit2	16 : 57.156665	-12.44	NA	High	Median
67581	Tbc1d23	16 : 57.168858	-12.43	NA	High	Median
71027	Tmem30c	16 : 57.266139	-12.33	NA	High	Median
12837	Col8a1	16 : 57.624256	-11.98	NA	High	Median
73379	Dcbld2	16 : 58.408426	-11.19	NA	High	Median
12892	Cpox	16 : 58.670208	-10.93	NA	High	Median
68146	Arl13b	16 : 62.793308	-6.81	NA	High	Median
19128	Pros1	16 : 62.854307	-6.75	NA	High	Median
72020	Zfp654	16 : 64.780347	-4.82	NA	High	Median
106143	Cggbp1	16 : 64.852001	-4.75	NA	High	Median
68942	Chmp2b	16 : 65.539133	-4.06	NA	High	Median
73569	Vgll3	16 : 65.815015	-3.78	NA	High	Median
19876	Robo1	16 : 72.027551	2.43	NA	High	Median
268902	Robo2	16 : 73.891976	4.29	NA	High	Median
54613	St3gal6	16 : 58.469742	-11.13	NA	Low	Low
106338	Nsun3	16 : 62.732444	-6.87	NA	Low	Low
68159	Stx19	16 : 62.814676	-6.79	NA	Low	Low
239857	Cadm2	16 : 66.655416	-2.94	NA	Low	Low
224344	Rbm11	16 : 75.592844	5.99	NA	Low	Low
69457	Tmem45a2	16 : 57.036967	-12.56	NA	NA	Low
66497	Cmss1	16 : 57.302	-12.30	NA	NA	Low
224250	Cldnd1	16 : 58.72791	-10.87	NA	NA	Low
67014	Riox2	16 : 59.47177	-10.13	NA	NA	Low
224291	Csnka2ip	16 : 64.47781	-5.12	NA	NA	Low
18736	Pou1f1	16 : 65.520629	-4.08	NA	NA	Low
224318	Speer2	16 : 69.856874	0.26	NA	NA	Low
52645	D16Ertd519e	16 : 70.616425	1.02	NA	NA	Low
751561	Mir691	16 : 74.34199	4.74	NA	NA	Low
102467647	n-TIaat1	16 : 75.434179	5.83	NA	NA	Low
320355	Lipi	16 : 75.540514	5.94	NA	NA	Low

Note: This list excluded the 25 olfactory receptor genes. ^∗^ indicates that the genes are expressed in hair cells, epithelial nonhair cells, or the cochlear duct. Expression data were extracted from the gEAR portal (https://umgear.org/). Expression value greater than 1000 is defined as high expression. NA: data not available.

## Data Availability

No additional data were used to support this study.
